# Understanding the Molecular Impact of Physical Exercise on Alzheimer’s Disease

**DOI:** 10.3390/ijms252413576

**Published:** 2024-12-18

**Authors:** Alba Cantón-Suárez, Leticia Sánchez-Valdeón, Laura Bello-Corral, María J. Cuevas, Brisamar Estébanez

**Affiliations:** 1 Faculty of Health Sciences, University of Leon, 24071 Leon, Spain; acants00@estudiantes.unileon.es; 2 Health Research Nursing Group (GREIS), University of Leon, 24071 Leon, Spain; lsanv@unileon.es (L.S.-V.); lbelc@unileon.es (L.B.-C.); 3 Department of Nursing and Physiotherapy, University of Leon, 24071 Leon, Spain; 4 Institute of Biomedicine (IBIOMED), University of León, 24071 Leon, Spain; mj.cuevas@unileon.es

**Keywords:** Alzheimer’s disease, amyloid β-peptide, mitochondrial dysfunction, molecular mechanisms, neurodegeneration, neuroglia, oxidative stress, physical activity, tau protein

## Abstract

Alzheimer’s disease is one of the most common neurodegenerative diseases, characterized by a wide range of neurological symptoms that begin with personality changes and psychiatric symptoms, progress to mild cognitive impairment, and eventually lead to dementia. Physical exercise is part of the non-pharmacological treatments used in Alzheimer’s disease, as it has been shown to delay the neurodegenerative process by improving the redox state in brain tissue, providing anti-inflammatory effects or stimulating the release of the brain-derived neurotrophic factor that enhances the brain structure and cognitive performance. Here, we reviewed the results obtained from studies conducted in both animal models and human subjects to comprehend how physical exercise interventions can exert changes in the molecular mechanisms underlying the pathophysiological processes in Alzheimer’s disease: amyloid β-peptide pathology, tau pathology, neuroglial changes, mitochondrial dysfunction, and oxidative stress. Physical exercise seems to have a protective effect against Alzheimer’s disease, since it has been shown to induce positive changes in some of the biomarkers related to the pathophysiological processes of the disease. However, additional studies in humans are necessary to address the current lack of conclusive evidence.

## 1. Introduction

Alzheimer’s disease (AD) is a neurodegenerative disorder that causes memory loss, impaired executive function, and personality changes amongst other neurological symptoms. AD is caused by synapse loss and neuronal atrophy, and it is characterized by the presence of amyloid plaques and neurofibrillary tau tangles (NFTs) [1,2].

AD is considered the primary cause of dementia, a heterogenous syndrome caused by a wide range of neurological diseases that results in a major decrease in cognitive function interfering with occupational, household, or social functions. AD, Parkinson disease, dementia with Lewy bodies, and vascular dementia are some of the most prevalent neurodegenerative diseases (NDs) [3,4]. NDs represent a serious global health issue, with a rising incidence rate and an estimated 57.4 million people currently living with dementia worldwide. This number is estimated to grow to 83.2 million in 2030, 116.0 million in 2040, and 152.8 million in 2050 [5,6].

NDs may have environmental, genetic, or complex aetiology and cause gradual neurodegeneration in several areas of the nervous system. The hallmark of this neurodegeneration is the loss of neurons and synapses, although it usually involves a variety of independent cell types [5,7]. Even though the fundamental mechanisms of NDs are quite complex and still under investigation, it is known that some genetic factors are associated with synaptic physiology and its alteration [8]. Synapses are the basic information processing units that enable a functional connection between two neurons. Through synaptic transmission, the brain receives and processes all the information, and these forms of transmissions can be both electrical and chemical. Synaptic organization is dynamic and sensitive; even slight changes can cause severe imbalances and an array of neurological symptoms [9,10,11].

There are a large number of treatment strategies for NDs that can be divided into two groups: pharmacological (the use of different drugs to reduce the production or toxicity of amyloid or tau, reduce neuroinflammation and neurodegeneration, or reduce neurological symptoms) and non-pharmacological interventions (lifestyle modifications to decrease disease progression) [12].

Among non-pharmacological interventions, the role of physical exercise (PE) is becoming more and more interesting. The positive impact of PE on brain function has long been discussed and several studies have shown the beneficial effects of PE on elderly populations such as the reduction in the risk of AD and dementia [13,14]. PE was demonstrated to delay the neurodegenerative process by increasing blood flow from the brain to the hippocampus, modulating neuroinflammation and enhancing neuroplasticity by modifying synaptic function and structure in various brain areas [14,15]. Since, in 2022, expected lifetime care costs for a person with dementia were USD 392,874, PE intervention seems to be a promising, cost-effective treatment to prevent and slow down the progression to dementia and cognitive impairment in older individuals who suffer NDs. Nevertheless, the biochemical and molecular mechanisms by which PE enhances cognition seem to be complex and still partly unclear [15,16,17].

It is essential to comprehend the molecular mechanisms through which PE exerts its effects to enhance health and slow neurodegeneration in AD. Therefore, the aim of this review was to explore the molecular mechanisms involved in using PE as an intervention for AD patients.

## 2. Alzheimer’s Disease

Estimating the global population with AD is rather difficult, as the distribution of people with the disease is highly inhomogeneous across the world. Some authors estimate that about 55 million people worldwide suffer from AD; however, this number typically refers to AD patients who present dementia symptoms, but if people in predementia stages are taken into account, the estimated number of individuals with AD may grow to 416 million, ranging between 327 million and 524 million [18,19,20]. The prolonged period of illness before death, which is often spent in a state of extreme reliance and impairment, greatly increases the public health effect of AD. In terms of disability-adjusted life years, AD was considered the sixth most burdensome disease or injury in 2016 [17].

In the future, it seems certain that the number of AD patients is expected to increase, mainly due to the aging of the population, given that age is one of the most relevant risk factors in AD. That might also be one of the reasons why AD is more prevalent in women, since they have an increased longevity compared to men [17,20,21].

**Figure 1:**
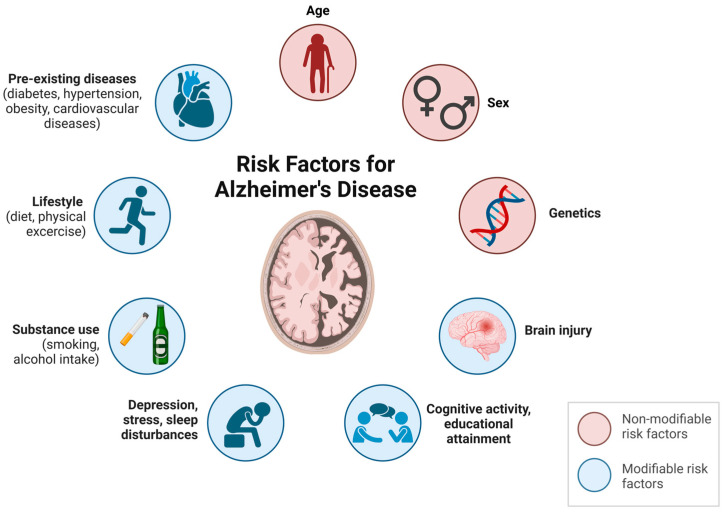
Non-modifiable and modifiable risk factors for AD. Age, gender, and genetics are considered non-modifiable factors in AD. Conversely, psychosocial factors (cognitive activity, educational attainment, depression, stress, or sleep disturbances), environmental factors (smoking, alcohol intake, diet, or physical exercise), and pre-existing diseases (diabetes, hypertension, obesity, or cardiovascular diseases) are considered modifiable factors in the development of AD.

Age and sex are part of the non-modifiable risk factors that could influence the development of AD, as well as genetic factors; it is known that the inheritance of particular mutations in genes like amyloid precursor protein (*APP*), presenilin-1 (*PSEN-1*), presenilin-2 (*PSEN-2*), and apolipoprotein E (*ApoE*) are associated with AD [22,23,24]. However, AD is likely to be multifactorial, with complex interactions between non-modifiable and modifiable risk factors. The latter includes psychosocial factors (educational attainment, cognitive activity, depression, stress, sleep disturbances, etc.), environmental factors (diet, PE, substance use, etc.), and the presence of pre-existing diseases such as diabetes, hypertension, dyslipidaemia, obesity, cardiovascular diseases, traumatic brain injury, or hyperhomocysteinemia (Figure 1) [22,24,25,26].

AD is characterized by a progressive disease continuum generally divided into different stages. The nomenclature of these stages may vary depending on the author, but essentially, in almost every staging system, the disease starts with preclinical AD as an asymptomatic phase with biomarker signs of AD, progresses to mild cognitive impairment (MCI) and/or mild behavioural impairment (MBI), and eventually concludes with AD dementia [27,28,29].

There are different AD diagnostic criteria that have evolved from requiring postmortem pathological confirmation for a definitive diagnosis to enable AD diagnosis to be made far earlier and with greater molecular specificity [30,31]. Two of the most recent and recognized AD diagnostic criteria are the ones proposed by the National Institute on Aging-Alzheimer’s Association (NIA-AA) and the International Working Group (IWG). Despite both sets of criteria including preclinical AD phases, the basis of an AD diagnosis continues to be clinical evaluation, focused on a clinical interview with the patient and a cognitive and physical examination, combined with pathological confirmation through tests like positron emission tomography (PET) or magnetic resonance imaging (MRI), to obtain a definitive AD diagnosis [31,32,33,34]. At present, AD cannot be diagnosed solely based on the presence of specific blood or genetic biomarkers. However, some authors emphasize the importance of early detection of AD in presymptomatic phases by identifying representative biomarkers of the disease. This would allow early therapeutic intervention with a higher opportunity of success since treatments would be carried out prior to the worst synaptic and neuronal damage. Additionally, the use of biomarker tests may be more affordable and accessible in clinical practice [35,36,37,38].

Neurological symptoms caused by AD are crucial for its diagnosis. The symptoms are diverse and change significantly throughout the continuum of the illness [24]. In early stages, symptoms frequently are misdiagnosed, incorrectly ascribed, or disregarded and they manifest years before receiving a clinical diagnosis of dementia. Since the neurons in brain areas involved in memory, language, and thinking are the first to suffer damage, changes in mood and sleep, heightened anxiety, impulsivity, depressive symptoms, apathy, withdrawal from social activities, communication difficulties, and visuospatial problems are some of the earliest AD symptoms [17,26,27]. Later, as the neurological damage becomes more severe, symptoms such as memory loss, confusion, or great behavioural and personality changes appear. This set of symptoms is known as MCI [28,31]. Ultimately, AD leads to dementia; in this stage, all mentioned symptoms worsen and become more evident and basic functions such as swallowing or walking are compromised, leading to major reductions in function and quality of life. These behavioural and psychological symptoms of dementia are linked to increased mortality and cause significant distress for families and caretakers [17,24,39].

Nowadays there is no effective cure for AD, and available treatment strategies only mitigate symptoms to improve quality of life [25]. Pharmacological management of AD mainly includes cholinesterase inhibitors (ChEIs) (donepezil, galantamine, and rivastigmine) and the N-methyl-D-aspartate (NMDA)-antagonist, memantine. Consensus guidelines and practice norms often recommend both therapies. Additionally, memantine and ChEIs work in complementary ways and may have cumulative effects. Aside from the mentioned drugs, there are other therapies that are currently being developed such as gene therapy, immunotherapy, or the use of peptidomimetics and nanoparticles. In AD patients, it is also important to identify and treat comorbid conditions that decompensate dementia and acknowledge the use of antipsychotics under specific circumstances [26,28,38,40].

Non-pharmacological interventions employed in AD do not alter the essential biology of the disease and are frequently used to preserve or enhance cognitive function, general quality of life, and the capacity to perform everyday living activities. Non-drug therapies also reduce behavioural and psychological symptoms like sadness, apathy, wandering, sleep difficulties, agitation, and hostility; this is why they are the main techniques utilized in practice to address agitation, aggression, and problem behaviours in general. Examples of non-pharmacological strategies include memory and orientation exercises, music- and art-based therapies, and PE [17,26].

It is yet unknown what causes the pathological alterations in AD. Although a number of theories have been proposed to explain AD, two are thought to be the primary causes: a change in the synthesis and processing of amyloid β-protein (Aβ) or a malfunction in cholinergic function. As there are various multiple and complex pathways affected by the disease, we can further extend these two theories to include the deficient brain use of glucose, oxidative stress, neuroinflammation, vascular anomalies, and harmful alterations in the mitochondria. Nevertheless, there is currently no accepted theory to explain the pathogenesis of AD [22,23].

The development of NFT, excessive accumulation of Aβ plaques, and neuronal loss are the primary pathological hallmarks of AD [28]. In a healthy state, Aβ is a small, water-soluble peptide that comes from the cleavage of the native APP by the enzymes α-secretase, β-secretase, and γ-secretase. If the processing of APP is disrupted, toxic oligopeptides are formed and deposited in neurological areas. Certain Aβ fragments are cytotoxic, especially to neurons. Aβ plaques (abnormally folded Aβ proteins) induce the production of oxygen radicals that are harmful to nerve cells. The primary cause of their toxicity is the dysregulation of calcium homeostasis caused by lipid abnormalities in neuronal cell membranes, which results in the death of the neurons [18].

Tau is a member of the family of microtubule-associated proteins, and it is a heat stable protein essential for the stability and assembly of microtubules. Normal tau can be found in neuron axons. In AD, modified hyperphosphorylated tau proteins are abnormally deposited in neurons, forming aggregates known as NFTs [41]. These tau forms are unable to maintain the structure of the cell. Additionally, NFTs affect regular cellular functions such as signal transduction, axonal transport, or synaptic transmission, which ultimately lead to cell degeneration [38]. To some authors, there exists a correlation between the accumulation of Aβ and the synthesis of NFTs; both would act in parallel, enhancing each other’s toxic effects. To other authors, the aggregation of the tau protein is a downstream effect of Aβ plaques [18,24].

**Figure 2:**
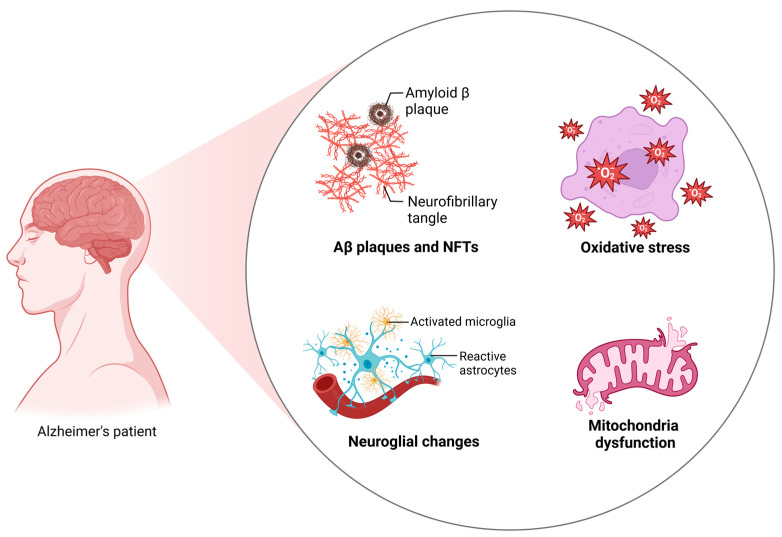
AD main pathophysiological mechanisms. The presence of amyloid plaques and NFTs in the brain of AD patients alongside changes in neuroglial cells, increased levels of oxidative stress, and mitochondrial damage and dysfunction are the main pathophysiological mechanisms present in AD.

In addition to Aβ plaques and NFTs, other mechanisms have long been implicated in the pathophysiology of AD, such as oxidative stress, glial changes, genetic changes, and mitochondrial dysfunction. All these pathophysiological mechanisms combined lead to inflammatory processes that affect numerous cells and proteins like astrocytes, microglia cells, cytokines, and chemokines. Eventually, the malfunction of these cells generates neuroinflammation, affecting the environment around neurons and causing their damage (Figure 2) [18,42]. The molecular mechanisms involved in the neuroinflammation produced in AD will be discussed further in the following sections.

## 3. Role of Physical Exercise on Alzheimer’s Disease Patients

Results from several studies have suggested that adults should engage in exercise and physical activity to mitigate the detrimental effects of aging on cognitive function [43,44]. Physical activity is any movement of the body made by the skeletal muscles that demands energy expenditure, while exercise is a subset of physical activity that is planned, structured, and repetitive with the ultimate or intermediate goal of enhancing or maintaining physical fitness. It was also proposed that PE causes deeper molecular changes in different biological processes than physical activity does [45].

In healthy people, many exercise modes, intensities, and durations were investigated. There are different types or modalities of exercise [46,47]: (1) cardiorespiratory or aerobic exercise, which includes the large muscles of the body moving rhythmically for extended periods of time; (2) resistance, strength, or anaerobic exercise, which makes the muscles work or hold against a weight or force; (3) flexibility exercise, centred on movements intended to maintain or increase a joint’s range of motion; and (4) neuromotor exercise, based on emphasizing balance, proprioception, coordination, and agility with the object of reducing the probability of falling.

Of these modalities, the most frequently discussed and investigated in the research setting are cardiorespiratory and resistance exercises. Another factor that affects research results is the length of the intervention, which can range from acute to continued training [46].

Long-term exercise training is known to delay the start of physiologic memory loss; this indicates that exercise may be an effective way to prevent age-related memory loss and neurodegeneration. Nevertheless, it is important to point out that late-onset exercise programs have also had beneficial effects in delaying the aging of the brain. During the early stages of AD, aerobic exercise, either by itself or in conjunction with cognitive stimulation, improves some aspects of brain function like memory, attention, and executive function [48,49].

Exercise interventions may help prevent AD from developing in the first place, but, according to a number of physiological mechanisms, they also slow the advancement of the disease once it has already been diagnosed [50,51]. PE seems to alleviate some of the symptoms of AD by improving the redox state in the brain tissue and having anti-inflammatory effects [52]. Exercise also enhances the release of the brain-derived neurotrophic factor (BDNF), which would support neurogenesis, synaptogenesis, and dendritogenesis, improving both brain structure and cognitive performance [53,54].

Some evidence has also shown that physical activity has a favourable impact on human hippocampus volume, preventing the volumetric declines linked to aging and preserving the synaptic plasticity processes that occur in this brain area [51]. Additionally, exercise affects central neurotransmitter release, which can result in positive brain changes [54]. Additionally, some risk factors like vascular dysfunction, obesity, or diabetes, linked to a poorer prognosis in AD patients, can be mitigated by exercise [45,50,51].

However, despite all the benefits that PE seems to have to delay the onset or progression of AD, it must be taken into account that PE interventions provide a significant challenge for individuals with AD since their condition gets worse as cognitive deterioration worsens [47]. In addition, several studies have shown that intense PE can increase the production of reactive oxygen species (ROS). Since oxidative stress is associated with the development of AD, intense or extreme PE may not be beneficial for AD patients [55]. Nevertheless, with the available evidence, it is not certain that high-intensity PE can result in levels of oxidative stress that may be detrimental or positive to health [55,56].

In this review, several studies, conducted in both animal models and human subjects, were analysed to understand how PE interventions may modify the molecular mechanisms present in AD. The results will be assessed in the following sections.

## 4. Alzheimer’s Disease and Physical Exercise Intervention: Molecular Mechanisms

### 4.1. Amyloid Precursor Protein and Amyloid β-Protein Pathology: Molecular Mechanisms

The amyloid plaques that can be found in the brains of AD patients are extracellular lesions mainly composed of aberrant accumulation and oligomerisation of the Aβ peptides, which are derived from the processing of transmembrane APP by different secretase enzymes [57,58,59].

APP is a type I transmembrane precursor glycoprotein. The structure incorporates a short intracellular cytoplasmic C-terminal tail, an intra-membranous segment, and a large extracellular glycosylated N-terminal domain [60]. The *APP* gene is situated on chromosome 21 and has 18 exons that can be alternatively spliced into multiple APP isoforms ranging from 365 to 770 amino acids. There are three main isoforms: APP695, APP751, and APP770, named according to the number of amino acids they contain [57,61,62]. While APP695 is expressed in neurons, APP 751 and APP 770 are found mainly in glial cells [63]. APP is synthesized by the endoplasmic reticulum and processed through the secretory pathway to be ultimately derived via the Golgi apparatus to the plasma membrane, where it mostly localizes [60,64].

The precise physiological mechanisms of APP are still not fully understood. Numerous authors have thoroughly reviewed the significance of APP for neuronal development, survival, and plasticity, as well as for neurogenesis, memory, synaptic mechanisms, cell cycle, and calcium metabolism. APP controls synaptogenesis, neurite outgrowth, and neuronal migration, among other aspects of neuronal development. Given the importance of APP during development, it stands to reason that APP expression would be closely related to neuron survival [61,65,66]. APP interacts with the potassium-chloride cotransporter (KCC2), which participates in the regulation of neuronal activity through the gamma-aminobutyric acid (GABA) neurotransmitter. This interaction is carried out mainly in the hippocampus, a region specially related to memory and learning. An APP deficiency would accelerate the degradation of KCC2, alter GABAergic inhibition, and lead to neuronal hyperactivity that can affect normal brain activity, including cognitive function and synaptic plasticity [67]. However, it is uncertain if APP is protective or harmful to neurons since it seems to be crucial in neuronal development and in the establishment and maintenance of the nervous system, while it is also capable of producing neurotoxic Aβ peptides as a result of its processing [61].

**Figure 3:**
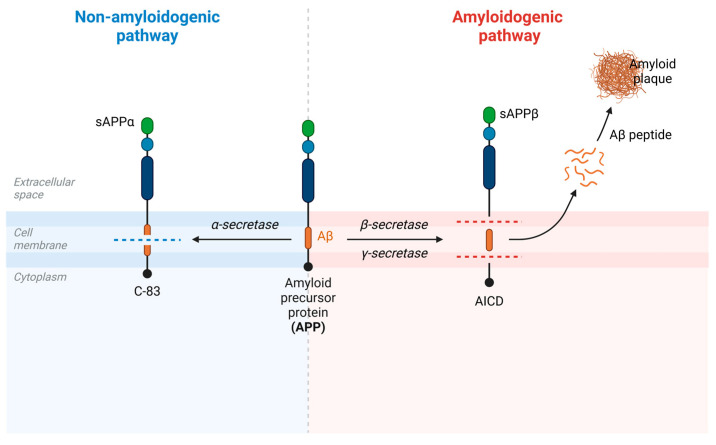
APP processing pathways. APP is cleaved by α-secretase and β- and γ-secretases into soluble fragments sAPPα and sAPPβ, respectively. While sAPPα does not lead to plaque formation (non-amyloidogenic pathway), Aβ peptides aggregate to form amyloid plaques (amyloidogenic pathway).

There are two ways of processing APP: through either the non-amyloidogenic pathway or the amyloidogenic pathway (Figure 3).

The non-amyloidogenic pathway is the most common one and it is initiated by α-secretase (ADAM10) releasing the membrane-attached C-terminal fragment 83 (C-83) and the N-terminal extracellular soluble APP domain (sAPPα). By cleaving the middle of the Aβ region, α-secretase prevents the further formation of Aβ. Then, γ-secretase intervenes, cutting C-83 and releasing the p3 molecule extracellularly and the APP intracellular domain (AICD) [62,65]. In the amyloidogenic processing pathway (enhanced in AD brains), β-secretase (BACE1) first cleaves APP at the N-terminus of the Aβ domain to produce a large secretory N-terminal soluble APP ectodomain (sAPPβ) and a membrane-bound C-terminal fragment 99 (C-99). Thereafter, the AICD and the intact Aβ peptide are released when γ-secretase cleaves C-99. Aβ peptides are composed of 37–43 amino acids; Aβ40 and Aβ42 are the most prevalent subtypes. While Aβ40 is considered to have a neuroprotective function, Aβ42 is generally regarded as neurotoxic due to its higher risk of protein aggregation and misfolding. Aβ40 and Aβ42 proportions vary in the development of AD, where an increase in Aβ42 occurs [59,68,69].

Aβ aggregates to form oligomers, protofibrils, fibrils, and plaques as a result of the dysregulation of Aβ levels due to an imbalance between the synthesis and clearance of these peptides. In the brain, Aβ is constantly being formed, but its aggregation and deposition mostly start in the entorhinal cortex and hippocampal regions [63,64]. Despite the early belief that amyloid plaques were the only harmful form of Aβ, research has revealed that Aβ oligomers (AβOs) can cause neuroinflammation, tau pathology, synaptic degradation, and adverse effects on neurons, among other toxic effects [70]. For example, AβOs are capable of interacting with N-methyl-D-aspartate receptors (NMDARs), causing an abnormal flow of Ca^2+^. This overstimulation leads to a deterioration in the synapse by decreasing the sensitivity of the receptors [71]. Furthermore, this anomalous flow of Ca^2^⁺ may also exert an influence on the formation of amyloid plaques, as it was demonstrated that Ca^2^⁺ ions play a pivotal role in modulating the structure, function, regulation, signalling activity, and self-association of certain amyloidogenic proteins, with the capacity to suppress or enhance the formation of amyloid fibrils. It was also demonstrated that specific Aβ proteins are capable of forming several types of amyloid fibrils depending on different conditions of the absence or presence of Ca^2^⁺ [72]. In addition, alterations in the concentration of Ca^2^⁺ and other ions in AD result in modifications to the ionic strength of the medium, which was also shown to influence the formation of different types of Aβ aggregates [73].

The sAPPα and sAPPβ domains are also obtained from the APP processing pathways. On one side, some evidence supports that sAPPα has neuroprotective qualities as it promotes the growth of neuronal progenitor cells and increases neuronal plasticity and neurogenesis, while APPβ is believed to regulate neuronal cell death and axonal pruning; in AD, the grey matter around the cerebral blood vessels and Aβ plaques has a greater concentration of sAPPβ [57]. On the other side, other authors maintain that both sAPP types are linked to neurite outgrowth and behave similarly, suggesting that sAPPβ is not always harmful to neuron function. Both domains would have a similar neurotrophic impact on neurons, promoting synapse formation by downregulating the GABAergic system and stimulating the glutamatergic system. However, sAPPα appeared to be more effective than sAPPβ [74].

The generation and aggregation of Aβ peptides are also linked to mutations in some genes like *PSEN-1*, *PSEN-2*, or *APP*. In the *APP* gene, 25 mutations were identified as harmful. *APP* mutations promote AD by either stimulating Aβ aggregation or enhancing the synthesis of the Aβ42 peptides but not by changing APP function. Approximately 200 distinct pathogenic variants for *PSEN-1* and *PSEN-2* were found to contribute to the development of AD. These two genes encode the proteins PSEN-1 and PSEN-2, and both proteins regulate the activity of γ-secretase. It was demonstrated that *PSEN* mutations impact γ-secretase function by destabilizing the enzyme–substrate complex. It was also proposed that mutations on *PSEN* cause pathological changes in mitochondrial metabolism, another cellular hallmark of AD [65].

#### PE Intervention Effect on APP and Aβ Molecular Mechanisms

In recent years, several studies were conducted in animal models to assess how PE can modify the molecular mechanisms behind APP processing and Aβ aggregation. These studies are usually performed on rodents with an induced AD (transgenic mice/rats or induced AD by injection of substances that generate a similar AD physiologic environment). Some studies showed a reduction in Aβ aggregation and/or number or Aβ plaques in those groups of animals with AD in which an aerobic or resistance PE training was performed, compared to sedentary groups, suggesting that PE interferes with Aβ deposition [75,76,77,78,79,80,81,82,83,84]. Additionally, in the studies carried out by Khodadadi et al., Zhang et al., Xia et al., and Zhao et al. aerobic PE specifically reduced Aβ42 peptide levels [78,79,82,85]. The study by Li et al. included a high-intensity interval training (HIIT) group, which showed a reduction in amyloid plaques in the hippocampus. This implies that high-intensity PE may be beneficial for AD [84].

Moreover, in some studies, molecules like PSEN-1 and BACE1 showed reductions in PE groups; these results suggest that PE attenuates the amyloidogenic processing of APP [75,79]. The same conclusion can be extracted from the results obtain by Lu et al. since levels of C-83 were higher than C-99 on the AD exercise group, providing evidence that aerobic exercise may mediate APP processing in favour of the reduction in Aβ deposits [83]. However, in all the studies mentioned above, physical training was scheduled. In a study conducted by Svensson et al., mice were subjected only to voluntary exercise. The results showed that there were no differences in Aβ levels between the exercise group and the sedentary one [86].

Since Aβ aggregation is a result of the imbalance between the synthesis and clearance of these peptides, Kodadadi et al. paid special attention to APP clearance pathways and discovered that the levels of the enzymes IDE and NEP and the LRP1 receptor, involved in the APP elimination process, were increased in the PE group, which would enhance the elimination of Aβ peptides [78].

Given that Aβ peptides can cause damage to synaptic sites, Liu et al. measured the concentrations of some presynaptic vesicle proteins: synapsin I, synaptobrevin-1, and synaptotagmin-1. No difference was found in synapsin I levels, but synaptobrevin-1 and synaptotagmin-1 levels increased in those mice submitted to resistance training intervention. Synaptotagmin-1 was implicated in synaptic vesicle endocytosis and is a crucial calcium sensor for synaptic vesicle exocytosis, while synaptobrevin-1 is essential for membrane fusion. Levels of PSD 95, an important postsynaptic structure protein, were also measured, and no significant changes were detected, which could mean that resistance exercise enhances presynaptic vesicle recycling and synaptic transmission, rather than postsynaptic structure [76].

Studies on the effects of PE on individuals with AD were also examined in this review. However, the evidence in humans is much more limited, especially when it comes to biomarkers associated with molecular mechanisms. Ornish et al. conducted a trial to check how intense lifestyle changes (aerobic PE, diet, supplements, etc.) could have an effect on patients with MCI AD. They observed an increase in the Aβ42/40 ratio in plasma in the group that underwent lifestyle changes. This might suggest that PE, alongside other lifestyle modifications, may exert a positive impact on Aβ accumulation [87].

Nevertheless, in other trials conducted in humans with AD, aerobic exercise interventions did not have any effect on biomarkers related to Aβ pathology (Aβ40, Aβ42, serum amyloid A (SAA), sAPPα, or APPβ). No significant difference was found either in plasma or in CSF [88,89]. Moreover, in a trial made by Vidoni et al. in patients with preclinical AD, aerobic PE did not have any impact on brain volume nor did it reduce the accumulation of amyloid peptides [90].

### 4.2. Tau Pathology and Neurofibrillary Tangle: Molecular Mechanisms

Tau is a microtubule-associated soluble protein heavily implicated in the development of AD. Tau protein is encoded by a single-copy gene, *MAPT*, located on chromosome 17q21. *MAPT* comprises 16 exons that are alternatively spliced on exons 2, 3, and 10 to generate 6 tau protein isoforms (3R0N, 4R0N, 3R1N, 4R1N, 3R2N, and 4R2N) in the central nervous system (CNS) and 6 additional isoforms in the peripheral nervous system (PNS) [91,92,93,94,95]. Tau is mostly expressed in neurons and in smaller levels in oligodendrocytes and astrocytes. Recently, tau was also detected in the tissue of the sigmoid colon and the human submandibular gland [96].

Tau protein is distinctly divided into different functional domains: an N-terminal projection domain, a proline-rich region (PRR), a microtubule-binding domain, and a C-terminal domain. The N-terminal domain length is dependent on the alternative splicing of exons 2 and 3, which encode acidic amino acids. The microtubule-binding domain is formed by three or four imperfect repeat sequences: R1, R2, R3, and R4 [94,96].

Being a microtubule-binding component, tau facilitates microtubule stability and polymerization. The 4R tau isoforms are more likely than the 3R tau isoforms to induce microtubule assembly, since tau connects to microtubules via C-terminal repeats within the microtubule-binding domain [97]. In addition to its main function stabilizing microtubules, it has an essential role in axonal transport. Tau is also known to be able to influence long-term synaptic depression, neuronal activity regulation, synaptic plasticity, neurogenesis, and iron export from neurons [96,98].

Tau binding to microtubules is mostly regulated by phosphorylation. Tau can be phosphorylated at multiple distinct locations by a variety of kinases, primarily ser/thr kinases (such GSK-3, CDK-5, MARK, PKA, CamKII, PKC, MAPK, JNK, or ROCK), and by tyrosine kinases (like Fyn or Abl) [94]. Tau phosphorylation was highly implicated in AD and widely researched, mostly due to the fact that hyperphosphorylated tau species are abundant in NFTs isolated from AD brains [93].

Essentially, tau hyperphosphorylation is the product of deregulated ser/thr kinases under pathological conditions. CDK-5 (cyclin-dependent kinase 5) and GSK-3β (glycogen synthase kinase 3β) are especially involved in the process [99]. GSK-3β and CDK-5 are activated to some extent by Aβ, causing these kinases to phosphorylate tau. At the same time, GSK-3 controls the synthesis of Aβ and the metabolism of APP and stimulates the death of neurons induced by Aβ. This is why some evidence suggests that there is a two-way interaction between Aβ and tau; however, it is also believed that Aβ and tau have unique and independent roles in AD [91,92,94,99].

It should also be taken into account that tau hyperphosphorylation can result from either excessive phosphorylation or diminished dephosphorylation. Tau is dephosphorylated by protein phosphatase 1 (PP1), PP2A, PP2B, and PP5. In AD patients, increased GSK-3β and CDK-5 expression and activity were observed, while PP1 and PP2A expression and activity were found to be decreased in certain brain areas [92,97].

**Figure 4:**
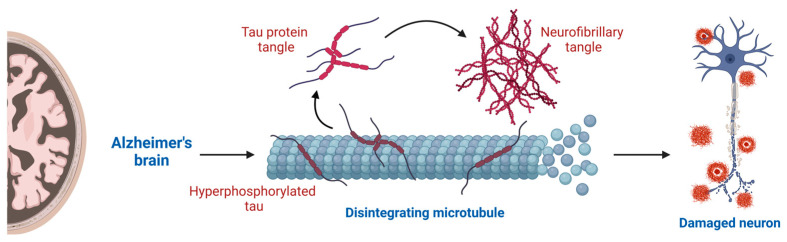
Tau pathology. Hyperphosphorylated tau cannot attach properly to microtubules, leading to microtubule disintegration and the aggregation of tau protein into paired helical filaments and NFTs.

Hyperphosphorylated tau may have pathogenic consequences in AD through various mechanisms: (1) Highly phosphorylated tau causes changes in microtubule stability. Tau experiences structural changes and detaches from microtubules since its affinity for tubulin is decreased or lost [92,98]. (2) Hyperphosphorylated tau promotes self-aggregation and progressively develops into paired helical filaments and NFTs. Moreover, inflammation accompanies the formation of these aggregates, which may help in their removal but may also exacerbate the pathogenic processes (Figure 4) [92,96]. (3) Synaptic transmission is disrupted by hyperphosphorylated tau, which is abnormally trafficked from axons to form aggregates in the somatodendritic compartment [92]. (4) Tau is often an optimal substrate for proteasomal degradation; thus, hyperphosphorylated tau aggregation inhibits tau turnover by reducing autophagy and subsequent protease cleavage [92,98].

#### PE Effect on Tau Pathology Molecular Mechanisms

In some of the studies analysed in this review, performed in animal models, lower levels of tau and phosphorylated tau were reported in those groups of animals in which a PE intervention was performed, compared to the control groups. This finding could indicate that training, both aerobic and strength-based, could indeed ameliorate AD tau pathology [75,76,77,81,83]. In addition, in two of the aforementioned studies, reductions were reported in some of the ser/thr kinases involved in the tau phosphorylation process in those animals that underwent an aerobic or resistance training program [75,76].

However, in the study conducted by Liu et al., it was shown that resistance exercise increased the levels of the GSK-3β kinase, which is closely linked to the formation of Aβ peptides. Alongside the activation of GSK-3β, the kinase Akt levels were also increased, suggesting that resistance exercise might reduce Aβ deposits and tau pathology by increasing the activity of the Akt/GSK-3β signal pathway (Akt would phosphorylate GSK-3β, preventing the last one to phosphorylate tau). The variations in both tau and APP biomarkers suggest that PE could induce feedback changes between both molecular mechanisms, avoiding not only Aβ aggregation but also NFT generation [76].

Hardly any evidence was found on how PE can affect the tau protein in patients with AD. In all the studies consulted, PE did not exert any type of influence on tau phosphorylation. No significant differences were observed between the sedentary and active groups in any of the biomarkers related to tau pathology, including p-Tau18 and t-tau, analysed in plasma [87,88] and CSF [89].

### 4.3. Neuroglia and Neuroinflammation in AD: Molecular Mechanisms

Glial cells, or neuroglia, accompany neurons across the nervous system, and they are the first cells to react to “stress” in the CNS. Astrocytes, microglia, and oligodendrocytes are the three main glial cell types found in the CNS [100]. Microglia are regarded as the main macrophages in the CNS that help in nerve development by phagocytosing and eliminating damaged neurons and synapses. The protective effect of microglia in the CNS is essential to maintain the homeostasis and promote the neuronal survival, differentiation, and synaptic formation related to learning [101,102].

To investigate the pathogenic development of AD, an accurate distinction of microglial phenotypes is crucial in the rapidly evolving brain environment. The microglia phenotypic types can be separated into two categories: activated phases and resting phase (M0).

**Figure 5:**
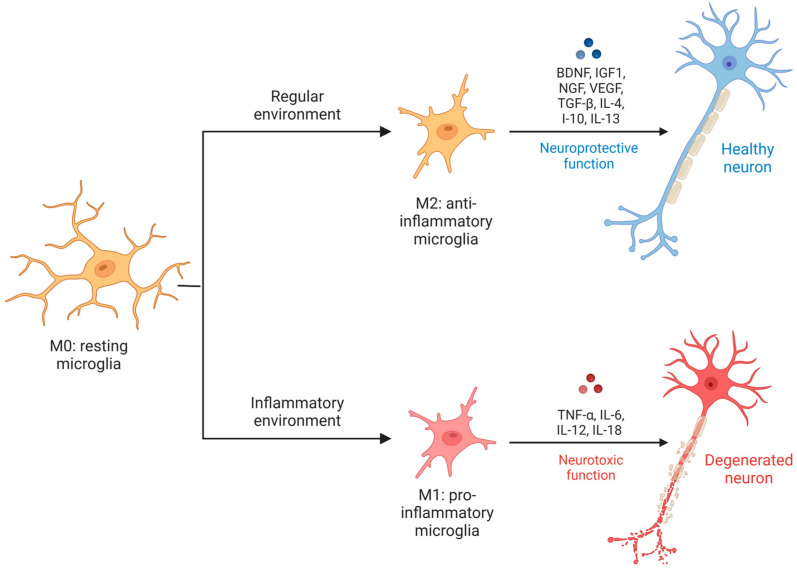
Microglia phenotypes. Depending on the stimuli received, resting microglia can change into activated phases with two different phenotypes: M2 or anti-inflammatory phenotype and M1 or pro-inflammatory phenotype.

At the resting phase, microglia are still constantly scanning, detecting alterations in the brain, and never rest. As changes in the brain microenvironment and glial cell are usually occurring, microglia can change from M0 to an activated phase (Figure 5) [101,103].

The activated phases can be divided into phenotype M2 and M1. On a regular basis, microglia exhibit the activated anti-inflammatory phenotype M2, which is characterized by the release of numerous trophic factors, including BDNF, insulin-like growth factor-1 (IGF1), nerve growth factor, vascular endothelial growth factor, transforming growth factor-β (TGF-β), and anti-inflammatory cytokines such as interleukin (IL)-4, IL-10, and IL-13. M2 microglia initiates tissue repair activities that include pathogen phagocytosis and the elimination of cellular debris and degenerated cells [101,104].

M2 microglia can be further classified into M2a, M2b, and M2c subclasses according to their unique pro-inflammatory cytokine profiles. Known as alternatively activated microglia, M2a microglia are linked to phagocytosis and neuroinflammatory resolution. Increased phagocytic and immunomodulatory activity is associated with M2b microglia, sometimes referred to as type II alternative activated microglia. M2c microglia, also known as acquired deactivated microglia, have anti-inflammatory properties [104].

In an AD context, inflammation persists and some enzymes like AMPK (AMP activated kinase) can induce microglia to change into the activated pro-inflammatory phenotype M1, which encourages inflammation and raises the levels of pro-inflammatory cytokines like tumour necrosis factor (TNF)-α, IL-6, IL-12, and IL-18 while also impairing phagocytosis. Activated microglia are particularly abundant near senile plaques in the AD brain, suggesting a critical role for them in the disease’s pathophysiology [101].

Aβ plaques affect the microglia as AD progresses. Aβ is capable of binding to a variety of microglia immune pattern recognition receptors (PRRs). The receptors for advanced glycation end products (RAGE), P2X7 receptor, toll-like receptors (TLRs), TREM2, class A scavenger receptor (SR-A1), and formyl peptide receptor-like-1 or Nformyl peptide receptor 2 (FPRL1) are a few examples [102,103,105].

The adhesion of Aβ to microglia through these receptors eventually results in compromised phagocytosis. Aβ induces microglia to release neurotoxic substances and pro-inflammatory cytokines, with TNF-α being one of the most relevant. TNF-α levels in the cerebrospinal fluid (CSF) or blood and CNS are very low in healthy persons, but they are markedly higher in the blood and CNS of AD patients. Aβ directly promotes the generation of TNF-α in microglia through the activation of the transcription factor NF-κB (nuclear factor kappa-light-chain-enhancer of activated B cells). The presence of TNF-α increases the expression of β- and γ-secretases, enhancing the generation of Aβ peptides [102]. NF-κB signalling is triggered through two different pathways: canonical and non-canonical. The canonical route is well researched and is essential for inflammatory responses, a crucial aspect of the development of AD. Hence, the persistent inflammation seen in AD is caused by the activation of NF-κB signalling and the subsequent release of cytokines and chemokines from microglia [106].

In AD, NF-κB also causes neurotoxicity by controlling specific microRNAs. It was demonstrated that NF-κB regulates microRNAs such miRNA-125b, miRNA-9, miRNA-155, miRNA-34a, and miRNA-146a. Among them, miRNA-125b is the most numerous in the human brain and is significantly upregulated in AD tissues. The miRNA-125b deregulates the complement system and amplifies the inflammatory response of the CNS. The miRNA-34a downregulates triggering receptors expressed in myeloid cells 2 (TREM2), which is essential for the microglial removal of Aβ [106].

Some of the genes that may be linked to inflammatory processes in AD are mostly expressed by microglia in the CNS. Among these genes are *TREM2*, *CD33*, *SIGLEC-3* (sialic acid-binding Ig-like lectin 3), *CR1* (complement receptor 1), *PLCG2* (phospholipase C gamma 2), and *INPP5D* (inositol polyphosphate-5-phosphatase D) [107].

Astrocytes are the most numerous brain non-neuronal cell type. Their functions include every cellular component of the CNS and are intimately linked to the nearby blood vessels and neurons. Pleomorphic activity is a characteristic of astrocytes that support CNS neurons by forming physical and chemical bonds with a variety of cytotypes. They represent the cellular interaction among the vascular compartment, brain microenvironment, and synaptic junction because of their responsiveness to external stimuli [108].

On certain pathological processes like AD, astrocytes become reactive, experiencing intricate and conflicting region-specific changes, such as functional, morphological, and cellular modifications. Reactive astrocytes can be divided in two subtypes: A1 (“pro-inflammatory”) and A2 (“anti-inflammatory”), which provide neurotoxic and neuroprotective effects, respectively [104]. What specifically determines the development of one phenotype over the other is not yet elucidated [108].

Astrocytes may cause atrophy or hypertrophy in AD. While astrocytes surrounding the senile plaques experience hypertrophy, those farthest from the amyloid deposits experience atrophy. Hypertrophied astrocytes accumulate around amyloid plaques, forming a physical barrier around them [104].

Astrocytes secrete complement proteins to carry out some of their regulatory tasks: whereas C8G-factor suppresses microglia activation and lowers inflammation, C3-factor controls the microglial activation and phagocytosis of cellular debris. C3 component is overexpressed in A1 pro-inflammatory astrocytes, indicating their potential neurotoxic effect. The absence of this molecule’s production by neuroprotective A2 astrocytes provides evidence of C3 toxicity. When Aβ plaques are present, astrocytes are stimulated to generate C3 [108]. Additionally, astrocytes induce neurodegeneration through NF-κB, which results in the production of pro-inflammatory cytokines, inducible nitric oxide synthase (iNOS), and Aβ42 accumulation [106].

#### PE Effect on Neuroglia Molecular Mechanisms in AD

Several studies conducted in animal models showed a reduction in the activation of microglia and astrocytes in the brains (frontal cortex and hippocampus) of those groups that performed aerobic [77] or resistance exercise [76,80]. Furthermore, Lu et al. and Zhang et al. analysed the phenotypes of microglia in both their investigations using specific markers. In both studies, an increase in the M2 phenotype and a decrease in M1 were seen in those animals with AD that followed a treadmill exercise intervention. Additionally, in Zhang et al.’s study, a reduction in the level of microglia associated with Aβ plaques was observed. These results could suggest that PE may cause microglia to adopt an anti-inflammatory phenotype [83,85].

On the other hand, Yang et al. evaluated the morphology of microglia, which showed fewer and shorter ramifications in the AD model and were reversed in those animals that underwent aerobic PE training [81].

Something crucial for the molecular analysis of microglia is the measurement of cytokine expression levels. The results of various investigations revealed that PE induces an increase in anti-inflammatory cytokines (IL-4, IL-10, TNF-β) while reducing pro-inflammatory ones (IL-6, IL-12, IL-18, TNF-α) [76,77,83,85]. The levels of cytokines secreted before and after physical training support the hypothesis of the shift from the M1 to M2 phenotype, implying a reduction in neuroinflammation [85]. In the same vein, a reduction in the anti-inflammatory cytokine IL-4 was determined in those animals that followed a resistance training, indicating that PE was able to reduce neuroinflammation [80].

Regarding the evidence of the effect of PE on the neuroglia of patients with AD, in some trials, such as the one conducted by Abd El-Kader et al., a decrease in the level of the inflammatory cytokines IL-6 and TNF-α was observed in the group that performed aerobic training [109]. In contrast, Jensen et al., in a similar study, detected higher levels of IL-6 in plasma in the subjects who underwent the PE intervention. This could be explained because IL-6 is produced in large quantities in healthy subjects with a rapid decline after the end of the exercise. In addition, IL-6 is also locally generated in the muscles exhibiting growth factor properties. This may suggest that exercise has similar effects on the IL-6 in AD as in healthy subjects. As well as the increase in IL-6, PE elevated the levels of sTREM 2 in CSF. This finding is difficult to interpret because TREM 2 can act both as a marker of microglia activation and as a molecule important in the clearance of Aβ [110].

De Farias et al., in their study in women with AD who underwent a functional training intervention, observed that, after training, IL-4 levels were higher and that the expression of NSE, a biomarker of neuronal damage, was reduced. It could be interpreted that PE exerted an anti-inflammatory influence on neuronal tissue. Nevertheless, none of the other analysed cytokines (IL-1β, TNF-α, and IL-10) presented significant differences pre- and post-intervention [111].

Regarding the study carried out by Delgado-Peraza et al., in which they obtained their results by analysing several biomarkers in neuron-derived extracellular vesicles (NDEVs) extracted from plasma, an increase in proBDNF and BDNF was found in the NDEVs of the patients who trained. These results support the idea that aerobic exercise can influence neuronal survival and regeneration and that NDEV cargo reflects the biochemical state of brain neurons and any dynamic changes to it [112].

In other studies, it was analysed whether PE could reduce markers of neuronal damage (GFAP, NfL, CRP, VILIP-1, YKL-40, and Ng), but in no case were significant differences observed between the sedentary and active groups [87,88,113].

### 4.4. Mitochondrial Dysfunction in AD: Molecular Mechanisms

Mitochondria supply the majority of the energy needs of cells by mitochondrial oxidative phosphorylation (OXPHOS), producing adenosine triphosphate (ATP) [114].

Many other biological processes depend on mitochondria, such as the regulation of intracellular calcium, bioenergetics, modulating the reduction–oxidation potential of cells, eliminating free radicals, and starting caspase-mediated cell death. In neurons, mitochondria control the level of Ca^2+^ needed for neurotransmission and the implementation of synaptic activities at the synaptic level in addition to regulating energy metabolism via ATP production and membrane potential maintenance [115].

In recent years, mitochondrial dysfunction has become an important hallmark of AD initiation and progression. Disrupted mitochondrial bioenergetics, increased oxidative stress, mitochondrial genomic stress, aberrant mitochondrial fusion and fission, abnormal mitochondrial axonal trafficking defects and aberrant mitochondrial distribution, impaired mitochondrial biogenesis, aberrant endoplasmic reticulum–mitochondrial interaction, impaired mitophagy, and impaired mitochondrial proteostasis are among the various mitochondrial abnormalities that were reported in AD patients [115,116].

**Figure 6:**
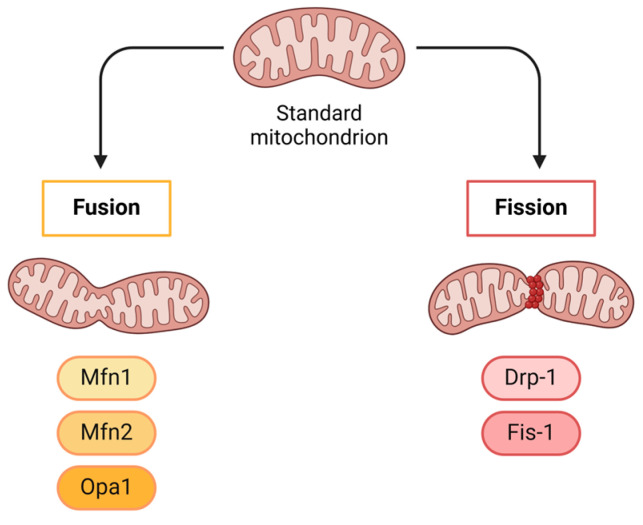
Mitochondria fusion and fission processes.

The growth, shape, distribution, and structure of mitochondria are all dependent on mitochondrial fusion and fission (Figure 6). Fusion is coordinated by three different GTPases: optic atrophy 1 (Opa1), mitofusin 1 (Mfn1), and mitofusin 2 (Mfn2). The mitochondrial fission process is mainly regulated by dynamin-related protein 1 (Drp1). Drp1 is mostly found in the cytoplasm, but when it is activated, it moves to the outer membrane to contract and split mitochondria through interaction with the mitochondrial fission factor and fission protein 1 (Fis1). The correct functioning of neurons depends on a healthy network of connections, which is ensured by the balance of mitochondrial fusion and fission. In AD, fusion and fission processes are deregulated: Opa1, Mfn1, and Mfn2 expression levels are reduced in brains of AD patients, while Fis1 expression is significantly elevated. Additionally, both hyperphosphorylated tau and Aβ interact with Drp1, which leads to an increase in the generation of free radicals. Thus, increased mitochondrial fragmentation, impaired mitochondrial transport to synapses, decreased synaptic ATP generation, and, finally, synaptic dysfunction result from the activation of Drp1 and Fis1 [117]. In response to the energy stress to which the mitochondria are subjected, structures called “mitochondria on a string” (MOAS) are formed. A MOAS is an intermediate mitochondria morphological phenotype that promotes mitochondrial stability and function. In AD, elevated levels of MOAS and mitochondrial fragmentation are often found due to the high energy stress [118].

Mitochondrial biogenesis is also impaired in AD. Peroxisome proliferator-activated receptor gamma coactivator 1-alpha (PGC-1α) is regarded as the master regulator of mitochondrial biogenesis and interacts with several transcription factors, including nuclear respiratory factor (NRF) 1 and NRF2, to coordinate and regulate energy metabolism and respiration. It was demonstrated that the levels of PGC-1α are significantly reduced in AD patients, leading to a disrupted mitochondrial biogenesis transcriptome signalling [119]. As a result, damaged mitochondria accumulate in neurons, diminishing cellular ATP levels and producing an excess of ROS, which can worsen mitochondrial damage [120].

Neurons are cells of highly demanding energetic processes, and their homeostasis is extremely dependent on mitochondrial health and mitophagy, which is a balance between the biogenesis of functional mitochondria and the autophagic destruction of the dysfunctional ones. There are two major types of mitophagy: PINK1 (PTEN-induced kinase 1)/Parkin-dependent and PINK1/Parkin-independent. Both are activated by different types of stimuli and proteins. In early AD stages, an increase in PINK1 was reported, and of Parkin at later stages, alongside an increase in mitochondrial content markers in both stages. This may suggest a defect in the initiation of the PINK/Parkin cascade and therefore the presence of impaired mitophagy in AD brains [114].

Some specific mitochondrial miRNAs localized in the mitochondria are also altered in AD. These miRNAs play important roles in mitochondrial function, as well as in various aspects of synaptic plasticity, activity, and neurotransmission. For instance, miR-132 is downregulated in AD and regulates the genes *PTEN*, *FOXO3a*, *P300*, *NOS1*, and *MMP-9*, which improve synaptic plasticity and neurotransmission. On the other hand, miR-34a upregulation in AD results in synaptic plasticity dysfunction via the modulation of VAMP2, SYT1, HCN, NR2A, and GLUR1 proteins [116,121].

#### PE Effect on Mitochondrial Dysfunction Molecular Mechanisms in AD

The GTPases Mfn1 and Fis1 are very important in the fusion and fission processes, respectively. In the study carried out by Yang et al., Mfn1 concentrations increased in those animals that exercised, while Fis1 concentration decreased. This suggests that aerobic exercise could help normalize the distribution and structuring of mitochondria, usually altered in AD. In the same study, PE also increased PGC-1α concentrations [81].

In the study by Li et al., reductions in Drp1 and Fis1 proteins, as well as an increase in Mfn1 and 2 and Opa1, were observed in both the aerobic and high-intensity training groups. These findings suggest that both types of exercise may exert a beneficial effect on the altered mitochondrial fission and fusion processes associated with AD [84].

Levels of PGC-1α were also measured by Liu et al., and they found an increase in this molecule in the group of rats submitted to resistance exercise. These findings suggest that PE, both aerobic and resistance, could help improve or reestablish mitochondrial biogenesis [76]. This hypothesis might be supported by the results obtained by Wu et al., in which PE increased the levels of NRF2, a transcription factor linked to mitochondrial biogenesis [77].

On the other hand, Lu et al. aimed to quantify the activity of ATP and cytochrome C oxidase (CCO) to determine whether PE could reduce mitochondrial dysfunction in AD. They found that ATP and CCO activity levels were increased in the PE group, suggesting that aerobic exercise may decrease mitochondrial dysfunction [83].

Since PINK1/Parkin-dependent pathway is one of the main mitophagy pathways altered in AD, Zhao et al. focused their study on investigating if aerobic exercise could regulate this pathway. The results showed that the abnormal PINK1/Parkin pathway was reversed, along with a reduction in the amount of mitochondrial damage in the exercised mice [82].

Mentions of biomarkers related to mitochondria in studies conducted in patients with AD are very scarce. Only the study by Delgado-Peraza et al. reported the levels of humanin, a mitochondria-derived peptide that suppresses neuronal apoptosis, preserves synapses, reduces inflammation, and supports glucose and oxidative metabolism. In this investigation, the NDEV levels of this peptide were elevated in patients who performed aerobic training, which would mean that exercise could ameliorate mitochondrial damage [112].

### 4.5. Oxidative Stress on AD: Molecular Mechanisms

The human body needs low amounts of oxidative stress to function properly, and these levels are useful for redox signalling and regulation. This is described as oxidative eustress. On the contrary, high levels of oxidative stress interfere with physiological functions and are known as oxidative distress. High levels of oxidative stress are usually due to an imbalance of ROS and the ability of the antioxidant system to effectively remove them. Over time, this imbalance causes impairment in redox homeostasis, resulting in a higher concentration of pro-oxidative species relative to antioxidants in the system [122,123].

The main types of ROS include hydroxyl radical (•HO), hydrogen peroxide (H_2_O_2_), superoxide (•O^2−^), and nitric oxide (NO). Because of their uncharged polarity, these ROS can pass through the cell membrane and cause lipid, protein, and DNA oxidation and protein glycosylation [122]. In AD, elevated levels of ROS cause damage in several cellular sites (biomacromolecules, sugars, lipids, proteins, and nucleic acids, among others), leading to advanced glycation end products, nitration, lipid peroxidation, and protein carbonyls. These damaged proteins can typically be eliminated by a number of degradative processes, such as autophagy, unfolded protein response pathways, and proteasomes. Nevertheless, each of these pathways is impaired in AD and MCI brains [123].

One of the characteristics of mitochondria dysfunction in AD is the excessive formation of ROS. In healthy organisms, mitochondria are responsible for 90% of the endogenous ROS because of the unavoidable electron leakage during the mitochondrial electron transport chain, which leads to the constant production of superoxide anion. The release of ROS by the mitochondria is usually compensated by the antioxidant system. However, in AD there is a decrease in the efficiency of the mitochondrial electron transport chain, in addition to a significant decrease in the activity of antioxidant enzymes such as superoxide dismutase (SOD), catalase (CAT), glutathione peroxidase, and heme oxygenase, resulting in the generation of an excess amount of ROS. Additionally, in AD there is also a reduced expression and/or activity of several key enzymes of oxidative metabolism, including α-ketoglutarate dehydrogenase complex, pyruvate dehydrogenase complex, and cytochrome oxidase [124,125].

Other molecules that play a central role in oxidative stress generation and defence are metal ions, in particular Cu, Fe, and Mn. Cu and Fe, whether free or loosely bound, are highly effective ROS production catalysts. These metal ions, such as Cu in SOD1 or Fe in CAT, are also found in the catalytic centre of antioxidant enzymes. In AD, Cu, Fe, and other metal ions accumulate in the amyloid plaques and can reach concentrations much higher than physiological levels. Aβ can be coordinated with Cu and Fe, and the resultant complex may directly contribute to the generation of ROS [126].

#### PE Effect on Oxidative Stress Molecular Mechanisms in AD

In some studies, in animal models, PE showed beneficial effects on oxidative stress. Yang et al. demonstrated that treadmill exercise significantly reduced the levels of ROS and protein carbonyls (protein damage marker) [81]. In addition, they also observed a decrease in malondialdehyde (MDA), a lipid peroxidation product, and an increase in SOD levels. The same results were obtained by Zhang et al. in relation to oxidative stress. The data collected imply that exercise may significantly reduce oxidative stress-induced cellular damage in regions like the cortex and hippocampus [81,85].

In line with the results achieved in the two studies mentioned above, Wu et al. also determined reductions in MDA and protein carbonyls levels in the group of rats subjected to PE [77].

In the study conducted by Li et al., a reduction in ROS and MDA and an increase in SOD and CAT enzymes were observed in both the high-intensity and aerobic training groups. These findings suggest that both types of exercise could be beneficial in reducing oxidative stress levels in AD [84]. Although some studies indicated that high-intensity physical exercise can lead to the release of ROS, the results of the study by Li et al. suggest that the HIIT protocol performed by the mice resulted in a significant reduction in oxidative stress levels when compared to the control group [84].

The evidence in humans regarding how PE affects the molecular mechanisms of oxidative stress, as in the case of mitochondrial damage or tau pathology, is very limited. De Farias et al. found decreased levels of carbonyl, CAT, and ROS, while levels of serum sulfhydryl and nitrite increased in the blood of patients that underwent functional training.

**Figure 7:**
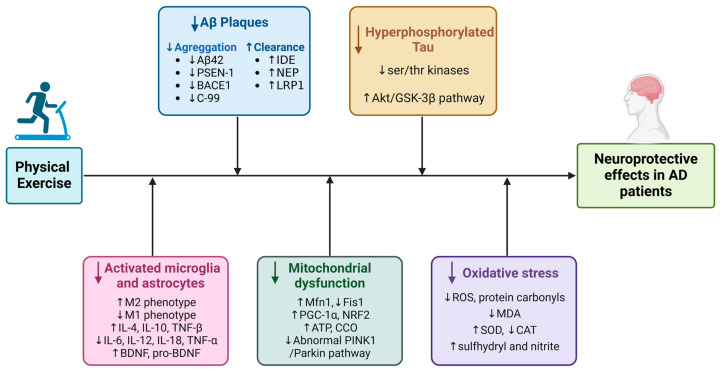
Schematic representation of the molecular mechanisms modified by PE in AD. Data extracted from the studies presented in Table 1 and Table 2.

This may indicate that exercise improved antioxidant capacity and lowered damaged biomarker levels, demonstrating that PE supports neuroprotection in AD patients through a variety of mechanisms (Figure 7) [111].

A summary of the studies conducted in animal models is presented in Table 1, while a summary of the studies in humans can be found in Table 2.

## 5. Conclusions

PE seems to promote a neuroprotective effect in patients with AD, since it was shown to induce positive changes in some of the biomarkers related to the pathophysiological processes of the disease, such as the reduced concentrations of Aβ peptides, tau protein, and pro-inflammatory cytokines, or increase the release of BDNF and anti-inflammatory cytokines. Nevertheless, because of its complexity, the complete picture of the molecular mechanisms behind the brain adaptations to exercise in AD patients is still unknown as well as difficult to comprehend. In addition, these modifications associated with the molecular mechanisms of AD are significantly more pronounced in studies conducted on animal models. This could be attributed to several factors, including the greater availability, ease, and accuracy of biomarker testing in animal models, as well as better monitoring of training interventions. Therefore, it is important to exercise caution when making broad assumptions, particularly when translating findings from animal models to humans.

Regarding the type of exercise, most of the available evidence refers to aerobic exercise. It would be interesting to perform additional investigation to gain further insight into how strength-based or resistance exercise and high-intensity exercise interventions might affect the molecular pathways in AD patients, since the available evidence is contradictory in some cases.

Future research may reveal a greater variety of AD biomarkers as well as easier and more accurate methods of detecting them in the plasma or CSF of human subjects. This, along with a deeper comprehension of the molecular mechanics underlying AD, may be the key to understanding how PE can positively influence the molecular pathways present in the disease.

## Figures and Tables

**Table 1:** Summary of animal model studies.

Reference	Study Population	Intervention	Results
Liu et al. [75]	Three-month-old mice: - APP/PS1 (*n* = 24) ○ TgC (*n* = 12) ○ TgE (*n* = 12) - WT (*n* = 24) ○ WTc (*n* = 12) ○ WTe (*n* = 12)	Treadmill exercise: - 30 min sessions, 5 days per week - 5 months	TgE vs. TgC: - ↓Aβ plaques and PSEN-1 - ↓sAPPβ and CTFs - ↓pTau (↓Thr205, Thr231, Ser396, and Ser404) - GSK-3α and GSK-3β inactivation
Lu et al. [83]	Sprague Dawley rats: - STZ (AD model) (*n* = 16) ○ STZc (n = 8) ○ STZe (*n* = 8) - Normal ○ NormC (*n* = 8) ○ NormE (*n* = 8)	Treadmill exercise: - 20 min sessions, 5 days per week - 4 weeks	STZe vs. STZc: - ↓C-99/C-83 ratio - ↓Aβ generation - ↓pTau - ↓M1 microglia phenotype markers and ↑M2 microglia phenotype markers - ↓IL-1β and TNF-α - ↑IL-4 and IL-10 - ↓Mitochondrial dysfunction (↑CCO activity and ↑ATP)
Wu et al. [77]	Sprague Dawley rats: - STZ (AD model) (*n* = 18) ○ STZc (*n* = 9) ○ STZe (*n* = 9) - Normal ○ NormC (*n* = 9) ○ NormE (*n* = 9)	Swimming exercise: - 1 h per day - 26 days Note: AD was induced on day 29 and rats were sacrificed on day 56	STZe vs. STZc: - ↓Aβ generation - ↓pTau - ↑Synaptophysin and spinophilin. - ↓Glial activation - ↑IL-4 and IL-10 - ↓TNF-α, IL-1β, IL-6 and IL-18 - ↑Antioxidant capacity (↑NRF2) - ↓Oxidative stress molecules (MDA and protein carbonyls)
Khodadadi et al. [78]	Eight-month-old Wistar rats: - Aβ (AD model) (*n* = 28) ○ Aβc (*n* = 14) ○ Aβe (*n* = 14) - Normal (*n* = 28) ○ NormC (*n* = 14) ○ NormE (*n* = 14)	Treadmill exercise: - Two running intervals of 15 min with 5 min break - 5 days per week - 4 weeks	Aβe vs. Aβc: - ↓Aβ42 and Aβ plaque formation - ↑IDE and NEP enzymes - ↑LRP-1
Zhang et al. [85]	Three-month-old mice: - APP/PS1 (*n* = 12) ○ TgC (*n* = 6) ○ TgE (*n* = 6) - WTc (*n* = 6)	Treadmill exercise: - 45 min sessions, 5 days per week - 12 weeks	TgE vs. TgC: - ↓Aβ40/42 - ↓Aβ-associated microglia - ↓M1 microglia phenotype markers and ↑M2 microglia phenotype markers - ↓TNF-α and IL-1β - ↑IL-10 and TGF-β - ↓Oxidative damage (↓MDA; ↑SOD)
Xia et al. [79]	Three-month-old mice: - APP/PS1 (*n* = 24) ○ TgC (*n* = 12) ○ TgE (*n* = 12) - WT (*n* = 24) ○ WTc (*n* = 12) ○ WTe (*n* = 12)	Treadmill exercise: - 45 min sessions, 5 days per week - 3 months	TgE vs. TgC: - ↓Aβ plaques - ↓Aβ40/42 - ↓BACE1 and PSEN-1 - Downregulation of GRP78 and PERK-eIF2α pathway (↓ER stress)
Hashiguchi et al. [80]	Six- to seven-month-old mice: - APP/PS1 (*n* = 29) ○ TgC (*n* = 15) ○ TgE (*n* = 14) - WT (*n* = 27) ○ WTc (*n* = 15) ○ WTe (*n* = 12)	Resistance exercise: - Climbing a ladder with a progressive load - 5 days per week - 4 weeks	TgE vs. TgC: - ↓Aβ plaques - ↓Activated microglia - ↓IL-6 and IL-4
Svensson et al. [86]	Nine- to twelve-week-old mice: - 5xFAD (*n* = 30) ○ TgC (*n* = 14) ○ TgE (*n* = 16)	Voluntary exercise: - Mice were caged with a running wheel. - 24 weeks	TgE vs. TgC: - = Aβ species and plaques - = Activated microglia - = Cytokine levels
Liu et al. [76]	Nine-month-old mice: - 3xTg (*n* = 15) ○ TgC (*n* = 7) ○ TgE (*n* = 8) - WT (*n* = 16) ○ WTc (*n* = 8) ○ WTe (*n* = 8)	Resistance exercise: - Climbing a ladder with a progressive load - Alternate days - 4 weeks	TgE vs. TgC: - ↑Synaptotagmin-1 and synaptobrevin-1 - ↓Aβ plaques - ↓pTau and Tau - ↓Activated microglia and astrocytes - ↓TNF-α and IL-1β - ↑IL-10, IL-6, and PGC1-α - ↑GSK-3β - ↓Phosphorylated ser/thr kinases
Yang et al. [81]	Two-month-old rats: - TgF344-AD (*n* = 25) ○ TgC (*n* = 12) ○ TgE (*n* = 13) - WT (*n* = 12)	Treadmill exercise: - 45 min sessions, 3 days per week - 8 months	TgE vs. TgC: - ↓Aβ deposition - ↓pTau - Preservation of synapses - ↑Mfn1, PGC-1α; ↓Fis1 - ↑SOD2; ↓ROS - ↓Oxidative damage (MDA, protein carbonyls) - ↓Activated microglia and astrocytes - ↓IL1-α, IL-1β, IL-3, IL-6, and TNF-α
Zhao et al. [82]	Three-month-old mice: - APP/PS1 (*n* = 18) ○ TgC (*n* = 9) ○ TgE (*n* = 9) - WT (*n* = 18) ○ WTc (*n* = 9) ○ WTe (*n* = 9)	Treadmill exercise: - 45 min sessions, 5 days per week - 12 weeks	TgE vs. TgC: - ↓Aβ plaques and Aβ40/42 - Reversed abnormal PINK1/Parkin mitophagy pathway. - ↓Damaged mitochondria
Li et al. [84]	Three-month-old mice: - APP/PS1 (*n* = 36) ○ TgC (*n* = 12) ○ TgHIIT (*n* = 12) ○ TgMICT (*n* = 12) - WT (*n* = 12) ○ WTc (*n* = 12)	HIIT protocol: - Treadmill 5 days per week - 12 weeks - 9 bouts of high-intensity running and 9 bouts of active recovery MICT protocol: - Treadmill, 30 min sessions, 5 days per week - 12 weeks	TgHIIT and TgMICT vs. TgC: - ↓Aβ deposition on hippocampus - ↓Drp1, Fis1 - ↑Mfn1, Mfn2, Opa1 - ↓ROS, MDA, H_2_O_2_ - ↑SOD, CAT

Abbreviations: 5xFAD, AD transgenic mice; APP/PS1, AD double transgenic mice; CTFs, C-terminal fragments; ER, endoplasmic reticulum; pTau, phosphorylated tau; STCc, STZ control group; STZ, streptozotocin; STZe, STZ exercise group; TgC, transgenic control group; TgE, transgenic exercise group; TgF344-AD, AD transgenic mice model; TgHIIT, transgenic high-intensity interval training; TgMICT, transgenic moderate-intensity continuous training; WT, wild-type rats/mice; WTc, WT control group; WTe, WT exercise group.

**Table 2:** Summary of human studies.

Reference	Study Population	Intervention	Results
Sewell et al. [88]	Cognitively impaired adults - Control (*n* = 32) - Moderate Intensity (*n* = 34) - High Intensity (*n* = 33)	Cycling on an ergometer: - 50 min, twice a week - 6 months - Moderate-intensity group cycled at a constant intensity. - High-intensity group cycled in intervals at high exertion combined with active recovery periods.	Plasma AD-related biomarkers pre- vs. post-intervention: - No significant differences were observed in Aβ42, Aβ40, Aβ42/40 ratio, p-Tau18, GFAP, and NfL.
Ornish et al. [87]	MCI AD patients - Control (*n* = 25) - Intervention group (*n* = 26)	Intensive multimodal lifestyle intervention: - Aerobic exercise at least 30 min/day and mild strength training exercises at least three times per week - 20 weeks Note: intervention also included changes in diet, stress management, group support, and the inclusion of supplements.	Intervention group vs. control group: - ↑Aβ42/40 ratio in plasma - No statistical differences were found in other AD relevant biomarkers (pTau181, GFAP, CRP, SAA, and C-peptide).
Delgado-Peraza et al. [112]	Mild to moderate AD patients: - Control (*n* = 47) - Exercise (*n* = 48)	Aerobic training: - 3 times per week - 16 weeks	Exercise group vs. control group: - ↑proBDNF, BDNF, and humanin on NDEVs
de Farias et al. [111]	Female AD patients (*n* = 15)	Functional training: - Coordination, agility, balance, strength, and endurance activities - 60 min, twice per week - 11 weeks	Blood AD-related biomarkers pre- vs. post-intervention: - ↑IL-4; =IL-1β, TNF-α and IL-10 - ↓NSE - ↑Serum sulfhydryl and nitrite - ↓Carbonyl levels, CAT, and ROS
Vidoni et al. [90]	Preclinical AD adults (elevated levels of cerebral amyloid) - Control (*n* = 39) - Exercise (*n* = 78)	Aerobic exercise: - 150 min per week - 1 year	Exercise group vs. control group: There were no differences in change measures of amyloid or brain volume.
Jensen et al. [110]	Mild AD patients - Control (*n* = 92) - Exercise (*n* = 106)	Aerobic exercise: - 60 min, 3 times per week - 16 weeks	Exercise group vs. control group: - ↑IL-6 (plasma) - ↑sTREM2 (CFS)
Jensen et al. [113]	Mild AD patients - Control (*n* = 26) - Exercise (*n* = 25)	Aerobic exercise: - 60 min, 3 times per week - 16 weeks	Exercise group vs. control group: - No significant differences in VILIP-1, YKL-40, NfL, and Ng were observed (CSF).
Abd El-Kader and Al-Jiffri [109]	AD patients - Control (*n* = 20) - Exercise (*n* = 20)	Treadmill aerobic exercise: - 40 min, 3 times per week - 2 months	Exercise group vs. control group: - ↓TNF-α and IL-6 (blood)
Steen Jensen et al. [89]	Mild AD patients - Control (*n* = 27) - Exercise (*n* = 29)	Aerobic exercise: - 60 min, 3 times per week - 16 weeks	Exercise group vs. control group: - There was no significant difference in Aβ38/40/42/, t-tau, p-tau, sAPPα, and sAPPβ concentrations (CSF).

Abbreviations: CRP, C-reactive protein; GFAP, glial fibrillary acidic protein; NfL, neurofilament light; Ng, neurogranin; NSE, neuron-specific enolase; VILIP-1, visinin-like protein-1; YKL-40, chitinase-3-like protein 1.
